# Treating Yourself in a Fairway: Examining the Contribution of Self-Compassion and Well-Being on Performance in a Putting Task

**DOI:** 10.3390/sports12110300

**Published:** 2024-11-05

**Authors:** Melanie R. Burgess, Diane E. Mack, Philip M. Wilson, Leah J. Ferguson

**Affiliations:** 1 Behavioural Health Sciences Research Lab, Department of Kinesiology, Faculty of Applied Health Sciences, Brock University, St. Catharines, ON L2S 3A1, Canada; 2 College of Kinesiology, University of Saskatchewan, Saskatoon, SK S7N 5B2, Canada

**Keywords:** self-compassion, well-being, mental health, sports performance

## Abstract

Researchers have advocated for greater insight regarding the contributions of psychological resources to sports performance. The purpose of this study was to examine the contributions of self-compassion and well-being to sports performance using a golf putting task. Male golfers (N = 87, M_age_ = 54.94; SD_age_ = 15.37 years) completed the Self-Compassion Scale—Athlete Version and the Warwick Edinburgh Mental Well-Being Scale immediately prior to the golf putting task which consisted of 15 consecutive putts from 7 feet on an outdoor practice green. Performance was assessed immediately following the putting task. Simple linear regression analyses showed that self-compassion did not predict ‘perceived’ (β = −0.20, *p* = 0.06; ƒ^2^ = 0.04) or ‘actual’ (β = −0.17, *p* = 0.11; ƒ^2^ = 0.03) performance. Similarly, well-being did not predict ‘perceived’ (β = −0.16, *p* = 0.15; ƒ^2^ = 0.03) or ‘actual’ performance (β = −0.01, *p* = 0.91; ƒ^2^ = 0.00). Overall, the conclusions from this study offer converging evidence that self-compassion and well-being may not impact putting performance in adult male golfers. Greater insight into whether, and if so under what conditions, self-compassion and well-being matter to sports performance warrants additional scrutiny.

## 1. Introduction

Researchers have long sought to understand the relationship between psychological resources and sports performance [1,2,3], with the full range of psychological factors yet to be unpacked. Within sports, self-compassion and well-being have been deemed important psychological resources linked to mental health, and the ability to overcome challenges and sustain careers [4,5,6]. Yet, it remains unclear if the benefits yielded by self-compassion and well-being extend to sports performance.

Self-compassion involves treating oneself with kindness as opposed to self-judgement, recognizing that we are not alone in our experience and balancing failure and pain with equanimity [7]. Current opinions imply that self-compassion benefits sports performance [4,6], yet this association has not been uniformly demonstrated. Notably, the findings of qualitative investigations, e.g., [8,9], have consistently endorsed athletes’ belief that self-compassion benefits sports performance. Conclusions from quantitative research linking self-compassion to sports performance, however, remain equivocal [10,11,12,13]. More specifically, in their retrospective investigation, Barczak and Eklund [11] reported a positive association between self-compassion and ‘perceived’ performance in adolescent swimmers. Killham et al. [14] reported a positive association between self-compassion assessed in the days prior to competition and ‘perceived’ performance following competition in female athletes competing in a variety of sports. Conversely, self-compassion was not statistically related to performance in athletes representing diverse sports when asked to complete a series of 40 m sprint tests [10], in gymnasts as rated by professional judges [12], or by the members of the U17 Swiss Cycling national team participating in a mountain time trial [13]. Given the equivocal nature of the published literature, whether self-compassion is associated with sports performance is unclear.

Well-being includes pleasant feelings and positive functioning related to the self and interpersonal relationships [15]. Collegiate and professional athletes have endorsed the belief that mental health/well-being affects their performance [16,17,18]. The nature of this relationship, however, may be complex. Higher well-being has been linked to lower ‘perceived’ performance in elite cyclists during the first, but not final, stage of a nine-day road race [19]. Well-being was positively associated with ‘actual’ performance during an outdoor, but not indoor, Track and Field Championships [20]. Further, using change in judges’ scores across one year, well-being was not associated with gymnastic performance [21]. Finally, Komáromi et al. [22] demonstrated a positive association between positive affect (i.e., emotional well-being) and ‘perceived’, but not ‘actual’ performance, in Motocross racers. In sum, the well-being–sports performance relationship appears to be fraught with considerable intricacy given the mixed evidence base. 

Intuitively, the link between self-compassion and well-being with sports performance appears plausible. Yet, considerable scope to investigate the relationship exists given the limited, and inconsistent, conclusions evident in the literature, e.g., [10,14,19,21]. This study was designed to address two additional gaps. With the exception of Komáromi et al. [22], the current knowledge about the contributions of self-compassion and/or well-being to performance in sports has relied exclusively on either ‘perceived’, e.g., [14,19], or ‘actual’, e.g., [10,13,21], measures of performance. Doorley et al. [23] argued that ‘perceived’ performance may be most relevant when exploring associations with subjective experiences such as self-compassion or well-being. Conversely, ‘actual’ performance is typical of how athletes are evaluated regardless of subjective appraisal processes. With the adoption of both ‘perceived’ and ‘actual’ performance, as in the present investigation, concerns expressed with the use of one marker of performance over the other are addressed. Further, the timing of data collection may hold implications for understanding the role of psychological resources relative to sports performance [24]. Given the differential associations reported with proximal, as opposed to more distal instrument administration [19], the timing of data collection may be implicated. 

Therefore, the objective of this investigation was to examine the unique and combined contributions of self-compassion and well-being to sports performance (This article is a revised and expanded version of a paper entitled “Treating yourself in a fairway: Examining the contribution of self-compassion and well-being on performance in a golf putting task.” Paper presented at the North American Society for the Psychology of Sport and Physical Activity, Toronto, ON, Canada, June 2023). Aligned with the existing research [11,14,17], when unique contributions were the focus, it was hypothesized that higher levels of self-compassion/well-being would predict higher ‘perceived’ and ‘actual’ sports performance. Researchers have not explored whether well-being contributes uniquely beyond self-compassion on ‘perceived’ and ‘actual’ sports performance. As such, when the combined influence of self-compassion and well-being on sports performance was under investigation, no formal hypotheses were advanced. 

## 2. Materials and Methods

### 2.1. Participants

Participant recruitment was guided by the following inclusion criteria: (a) Age (≥18 years, (b) self-identify as a golfer, (c) able to read/write English, and (d) consent to participate. The target sample size of 77 was informed by a priori power analysis using G*Power (version; 3.1.9.7) [25]. To determine sample size, an r = 0.28 was drawn from Barczak and Eklund [11] paired with a fixed probability value (*α* = 0.05) and statistical power (*β* = 0.80) The role of self-compassion relative to sports performance guided the a priori power analysis as this was the primary question under investigation in this study. The a priori power analysis was used based on ‘perceived’, not ‘actual’, performance given the timing of data collection. Insights regarding the role of self-compassion relative to ‘actual’ sports performance (i.e., [10,13]) were published following the data collection for this study. 

Eighty-seven male golfers (M_age_ = 54.94 years; SD_age_ = 15.37 years) provided consent to participate (see Table S1). Upon initial screening, an imbalance between self-identified males (n = 92) and females (n = 5) providing informed consent was evident. One potential reason for fewer women participating in the study may be due to the scheduling of the data collection which took place only on days when Men’s leagues were scheduled to play on the course where the putting green was used. Consequently, an examination of differences between the male and female participants was undertaken and reported (see Supplemental Table S2 and Figures S1–S4). The female participants were subsequently removed from consideration in this study. Most participants self-identified as Caucasian (n = 77; 88.5%) and considered themselves ‘recreational’ golfers (n = 77; 88.5%). Twenty-one golfers indicated they were a member of Golf Canada with an average handicap of 9.93 (SD = 6.31). The average rounds of golf played in 2022 prior to the data collection was 27.73 (SD = 19.53).

### 2.2. Procedure

Study procedures were reviewed and cleared by a university-based Research Ethics Board (REB File 21-315) and the study protocol was subsequently pre-registered (https://osf.io/8kmtz accessed on 2 August 2022). Following pilot testing (see Supplemental File), a multi-pronged recruitment strategy was adopted. Following the provision of informed consent, the participants arrived at the practice green and were asked to complete the demographic questionnaire followed by instruments assessing self-compassion and then well-being. The participants were then familiarized with the putting task using a standardized script to minimize the likelihood of introducing between-group effects within the data collection process. The participants then completed the golf putting task and the total number of putts holed was recorded. Regardless of the putting outcome, the ball was removed to avoid interference with the subsequent putts. Once completed, the participants were administered instruments to assess ‘perceived’ performance and challenge.

### 2.3. Instrumentation

Demographics: The participants were asked to complete items designed to assess general (e.g., age and self-identified ethnicity) and golf-related demographics. The golf-related demographic items assessed information about the golfer’s skill level and frequency of play in the current golf season. Three single-item putting questions were included (i.e., “How important is putting to golf performance?”, “How important is putting to your golf performance?”, and “How important is putting to you as a golfer?”) on a 5-point Likert scale ranging from 1 (“Not at all Important”) to 5 (“Extremely Important”).

Self-Compassion: Modified from Neff [26], the athlete version of the Self-Compassion Scale (SCS-AV) [14] was adopted. The 26-item SCS-AV assesses both the positive and negative aspects of self-compassion with response options ranging from 1 (“Almost Never”) to 5 (“Almost Always”) across six subscales: Self-kindness (5 items; sample item: “I’m tolerant of my own athletic flaws and inadequacies”); Common humanity (4 items; sample item: “I try to see my failings as part of the sport experience”); Mindfulness (4 items; sample item: “when something upsets me in my sport I try to keep my emotions in balance”); Self-judgment (5 items; sample item: “when times are really difficult in my sport, I tend to be tough on myself”); Isolation (4 items; sample item: “when I fail in my sport, I tend to feel alone in my failure”); and Over-identification (4 items; sample item: “when something upsets me in my sport I get carried away with my feelings”). The items were completed following the instructional stem “Please read each statement carefully before answering. Indicate how often you behave in the stated manner using the following scale.” For this investigation, the word ‘athlete’ was changed to ‘golfer’ and ‘sport’ to ‘golf’ to reflect the sample and performance task (sample item: “When things are going badly for me in golf, I see the difficulties as part of life that everyone goes through”). After reverse scoring the negatively worded items, the SCS-AV responses were calculated consistent with Neff [7]. Higher scores represent greater self-compassion in golf. Evidence for the validity and reliability of SCS-AV scores in athlete samples has been reported [14].

Well-Being: Well-being was measured using the 14-item Warwick Edinburgh Mental Well-being Scale (WEMWBS) [15] which assesses the extent to which an individual is feeling (sample item: “I’ve been feeling optimistic about the future”) and functioning (sample item: “I’ve been able to make up my own mind about things”) well. The instructional stem was altered to reflect global well-being to be consistent with that of the SCS-AV [14]. The response options ranged from 1 (“None of the Time”) to 5 (“All of the Time”). A total well-being score was computed by adding each item and dividing by 14 with higher scores corresponding to higher well-being. The score validity and reliability of the WEMWBS have been documented in athletic samples [27,28]. Approval to use the WEMWBS for the purpose of data collection for this study was secured on 28 April 2022.

‘Perceived’ Performance: The participants were asked to provide an appraisal of the quality of their golf putting performance. Following the instructional stem, “Considering your golf putting performance today…” the participants responded to the item, “Was it a good or bad performance for you?” on a 5-point Likert scale ranging from 0 (“Very Bad”) to 4 (“Very Good”). This single-item indicator has been used in other sports psychology research [11,29]. 

‘Actual’ Performance: The total number of putts holed out of 15 served as the measure of actual golf putting performance [30]. Putts holed received a score of ‘1’ while all the other putts received a score of ‘0’. Higher scores on the putting task indicate better ‘actual’ performance.

Challenge: Upon the completion of the putting task, the participants were asked to complete a single-item measure of task challenge: “How challenging was the putting task you performed today?” This descriptive item was measured across a five-point scale with response options ranging from 0 (“Not at All Challenging”) to 4 (“Very Challenging”).

### 2.4. Golf Putting Task

Environmental conditions may impact green speed and putting performance [31]. To address such concerns, conditions from Environment Canada were documented on the days when data were collected. The speed of the green was assessed using a stimpmeter prior to each session with readings taken as an average of three trials. Insight into the environmental conditions and green speed during the data collection can be found in the Supplemental File.

Guided by Turner et al. [30], the putting task was conducted using an outdoor practice putting green to enhance ecological validity. The flattest part of the green was used as determined by stimpmeter readings (i.e., the golf ball rolled approximately the same length from both directions). While the participants used their own putters, the type of golf ball was standardized (Titleist Pro V1) across the study participants. The length of the putt was set at 7 feet as Professional Association Golfers have a 50% chance of making a putt from this distance [32]. The participants first completed 6 familiarization putts that did not count towards their ‘actual’ performance. The next 15 performance putts were recorded by one researcher. The putts were completed from the same location on the green and remained uniform throughout the study. The researcher stood close to the hole, out of sight, and removed the ball once putted. The participants were entered into a draw for a 1 in 7 chance to win a $50 gift card. The study procedures were consistent with the regional Public Health COVID-19 protocols at the time of data collection.

### 2.5. Data Analyses

Data were analyzed using IBM^®^ SPSS (Version 28). Data were first screened for missing values, normality, outliers, and conformity with assumptions for relevant statistical tests. Second, internal consistency score reliability estimates for each multi-item instrument were calculated using coefficient alpha (α) [33] and coefficient omega (ω) [34]. Third, descriptive statistics and one-tailed bivariate correlations (Pearson r) for all the study variables were calculated. Fourth, a series of simple linear regression analyses to examine the ability of self-compassion or well-being to predict ‘perceived’ and ‘actual’ sports performance were conducted. Finally, separate hierarchical linear regression analyses were conducted to examine the joint contributions of self-compassion and well-being to predicting sports performance.

Standardized beta-coefficients (β) were used to evaluate the strength of the relationship between self-compassion/well-being and sports performance [35]. β coefficients can be affected by the magnitude of the covariance between explanatory variables [35,36] combined with the strength of the relationship between self-compassion and well-being (e.g., [10,37]). Therefore, additional coefficients were calculated to facilitate interpretation for the hierarchical regression analyses. First, structure coefficients (r_s_) were computed to examine the strength and direction of the relationship between individual explanatory variables and performance. r_s_ are less susceptible to contamination from suppressor effects or collinearity [38]. Second, the Relative Pratt Index (RPI) was calculated in each model to represent the proportion of variance attributed to self-compassion and well-being as a complementary measure of predictor importance [39]. A variable is meaningful when the RPI value is greater than 1/(2* the number of predictors) [40]. Any RPI value in this investigation greater than 0.25 was considered meaningful. For all inferential statistics, 95% confidence intervals (95% CI) and estimates of effect size were calculated and then interpreted. Consistent with Cohen [41], the f^2^ values of 0.02, 0.15, and 0.35 represent small, moderate, and large effect sizes for regression analyses.

## 3. Results

### 3.1. Preliminary Analyses

Eighty-nine participants provided informed consent. Two participants were deemed non-responders and removed from the subsequent analysis. The final sample size retained for analysis was 87. Non-response errors were observed on five of twenty-six SCS-AV items with no more than two participants (2.23%) failing to provide a response to that individual item. Little’s [42] test (*χ*^2^ = 165.60, df = 130, *p* = 0.17) implied that SCS-AV data may be missing completely at random. Non-response errors on four of fourteen WEMWBS items were observed with no more than one participant (1.11%) failing to provide data for any one item. Little’s [42] test (*χ*^2^ = 82.66, df = 38, *p* < 0.01) implied that WEMWBS data may not be missing completely at random. The missing values were replaced using an expectation–maximization algorithm before further analyses. 

### 3.2. Reliability Estimates, Descriptive Statistics, and Bivariate Correlations

The estimates of score reliability for the SCS-AV and WEMWBS item responses were α = 0.87; ω = 0.75 and α = 0.92; ω = 0.92, respectively, for the data provided. The average global score on the SCS-AV was 3.43 (SD = 0.55) and 3.87 (SD = 0.60) on the WEMWBS. On average, the perceptions of the importance of putting approached “extremely important” to (M > 4.50). The study participants averaged 7.86 (SD = 3.10; Range = 1.00–15.00) putts holed with ‘perceived’ performance approximating the mid-point of plausible response options (M = 2.56; SD = 1.10). The study participants typically rated their ‘perceived’ performance as “neither good nor bad”. In terms of the challenge of the putting task, the participant ratings approached the mid-point of the possible response options (M = 1.80; SD = 1.01). Bivariate correlations with 95% CIs are reported in Table 1. One participant was deemed an outlier based on the WEMWBS scores (z = −4.88), which on visual interpretation was deemed an influential, as opposed to leverage point, and removed from the subsequent analyses involving the WEMWBS scores.

### 3.3. Regression Analyses Predicting ‘Perceived’ and ‘Actual’ Sports Performance

No other assumptions of regression analyses were violated beyond the identification of the solitary outlier previously identified. Table 2 presents the results of the regression model analyses. The following observations were made when self-compassion was used to predict sports performance: (a) ‘perceived’ (F(1,85) = 3.42, *p* = 0.07; R^2^_adj_ < 0.03; f^2^ = 0.04); (b) ‘actual’ (F(1,85) = 2.46, *p* = 0.12; R^2^_adj_ = 0.02; f^2^ = 0.03). When well-being was used to predict sports performance, the following results were noted: (a) ‘perceived’ (F(1,84) = 2.53, *p* = 0.15; R^2^_adj_ = 0.01; f^2^ = 0.03); (b) ‘actual’ (F(1,84) = 0.01, *p* = 0.91; R^2^_adj_ = 0.00; f^2^ = 0.00). Self-compassion and well-being displayed negative regression coefficients in all the models. Therefore, higher scores of self-compassion (or well-being) pre-putt were associated with lower sports performance. The effect size estimates ranged from negligible to small [41].

To examine the combined effects of the explanatory variables in this study, self-compassion was entered at step one and well-being entered at step two of each hierarchical regression model (see Table 3). When ‘perceived’ performance was the response variable, the total variance accounted for was 2.60% (F(2,83) = 2.13, *p* = 0.13; R^2^_adj_ = 0.03; f^2^ = 0.03). The total variance accounted for when ‘actual’ performance was the response variable was 1.30% (F(2,83) = 1.56, *p* = 0.22; R^2^_adj_ = 0.01; f^2^ = 0.01). Well-being predicted no unique variance beyond the contributions of self-compassion to sports performance in either hierarchical regression model. The effect sizes ranged from negligible to small [41]. The interpretation of the RPI values demonstrated that self-compassion was a meaningful predictor of ‘perceived’ and ‘actual’ performance. Well-being was a meaningful predictor variable in the regression model for ‘perceived’ performance only given the RPI values. 

## 4. Discussion

Undeniably, sports researchers are invested in understanding the psychological factors that hold direct links with performance [1,2,3]. Self-compassion and well-being have been identified as potential resources to support performance in sports [4,11,16,19]. Despite such claims, support for direct relationships between self-compassion and/or well-being with sports performance has been equivocal [8,9,10,11,14,16,17,22] and therefore, warrants further investigation. The purpose of this study was to examine the contributions of self-compassion and well-being to sports performance using a golf putting task. Neither self-compassion nor well-being were predictors of ‘perceived’ or ‘actual’ performance among male golfers. Further, well-being did not predict unique variance beyond the contributions of self-compassion for either ‘perceived’ or ‘actual’ sports performance. Based on these observations, continued investigation into whether self-compassion and well-being are associated with sports performance appears justified. 

Neither self-compassion nor well-being in the present investigation predicted ‘perceived’ or ‘actual’ sports performance with negligible-to-small effects observed dependent on analysis. While the overall regression equation models did not differ statistically from the null, the direction of the relationship between the explanatory and response variables was negative. This finding suggests that higher self-compassion/well-being was associated with lower sports performance. One explanation may be that golfers with lower putting ability displayed higher self-compassion at baseline. Researchers have noted that self-compassion may help athletes regulate emotions in response to poor performance in sports [9,23]. It is plausible that athletes may enter sports tasks with higher levels of self-compassion because they have a sense of their own ability and anticipate lower performance. Extending to well-being, an inverse relationship with ‘perceived’ sports performance has been reported [19]. In their examination of individual athlete profiles, Komáromi et al. [22] reported that increased well-being post-competition was associated with performance that was rated as ‘better’, ‘as’, and ‘worse’ than expected. The above contributes to the noted intricacy of this relationship while simultaneously reinforcing that for some athletes, higher well-being scores may be negatively linked to performance. Researchers may wish to examine the utility of these claims as the ultimate test of these issues. 

Self-compassion has been examined in tandem with self-criticism [10,14], self-esteem [10], and various psychological skills/resources including self-talk and mental toughness [13] to investigate their contributions to sports performance. In this study, well-being did not predict unique variance beyond self-compassion for either performance measure. The interpretation of the calculated RPI values revealed self-compassion to be a more important predictor of performance than well-being. Researchers are encouraged to continue their investigation into the range of psychological resources that collectively underpin sports performance. 

There remains considerable scope to investigate whether (and if so for whom and when) self-compassion and well-being are linked with sports performance. Challenges associated with integrating conclusions across the literature are compounded by the use of different instruments to measure self-compassion and well-being by researchers. Extending beyond this, the characteristics of the sample and the nature of the sporting task may be important considerations. Most of the research linking self-compassion and/or well-being with sports performance has used either female or mixed-gender participants [8,14,18,21]. Yet, with the notable exception of Röthlin et al. [13] and Komáromi et al. [22], the investigations of this relationship using quantitative data in male athletes exclusively are limited. Researchers may want to further investigate whether gender moderates the relationship between self-compassion and/or well-being and sports performance. The literature informing the present investigation has typically been examined in adolescent [11,13] and young adult samples [8,10,14] which is contrasted against the average age of those providing data in this study. The present investigation addressed calls for the need to examine self-compassion across the breadth of a sportsperson’s lifespan [43]. Finally, the competitive level of the athlete may hold implications for the self-compassion/well-being–sports performance relationship. The majority of participants in this study considered themselves to be ‘recreational’ compared to more elite athletes studied in other investigations [13,14,22]. In recognition of this possibility, the findings of this study are comparable to Alipour Ataabadi et al. [10] who sampled athletes competing predominantly at the recreational and local levels. Differences in the nature of the sporting task (e.g., putting in golf vs. sprint performance vs. endurance performance) may also influence the relationship between self-compassion and/or well-being with sports performance. These reasons are currently speculative.

Although this study reports findings with implications for sports psychology research, select limitations warrant attention. This study used a cross-sectional research design with non-probability (purposive) sampling to recruit participants which limits the external validity of the study findings [44]. Researchers may want to use longitudinal research designs, employing multiple assessments (n > 3 time points), given that variability in well-being has been reported in athletes [22,45]. Data collected at multiple time points can be used to evaluate changes in the relationship between self-compassion and/or well-being relative to variability in sports performance over time. Finally, the practical implications of the study findings are difficult to assess given its focus and remain speculative. 

## 5. Conclusions

In conclusion, the psychological resources of self-compassion and well-being were not associated with ‘perceived’ or ‘actual’ sports performance in adult male golfers completing an ecologically valid golf putting task. It may be that the characteristics of self-compassion including responding to difficulties in life by being kind to oneself, being mindful of ongoing experiences, and recognizing that difficult times are part of the shared human experience may be more salient for those of lower ability in sports. Given that well-being was not meaningfully linked with sports performance, feeling and functioning well may not be linked with sports performance in the short term. Further contributions to knowledge including greater insight detailing for ‘whom’, ‘when’, and/or ‘how’ self-compassion and well-being impact sports performance warrants additional scrutiny. 

## Figures and Tables

**Table 1:** Bivariate correlations and 95% confidence intervals between study variables.

Variable	1	2	3	4
1. Self-Compassion	−			
2. Well-Being	0.35 (0.15, 0.52)	−		
3. ‘Perceived’ Performance	−0.20 (−0.39, 0.01)	−0.16 (−0.36, 0.05)	_	
4. ‘Actual’ Performance	−0.17 (−0.36, 0.04)	0.04 (−0.17, 0.25)	0.71 (0.59, 0.80)	_

Note: All r’s expressed are bivariate (Pearson) coefficients. Information in brackets is the 95% CI spanning the point estimate for each bivariate correlation. The sample size was *N* = 87 between self-compassion and performance. When the well-being scores were included in the analysis, n = 86. r values > |0.18| are statistically significant (*p* < 0.05) using a one-tailed null hypothesis significance test.

**Table 2:** Simple linear regression analyses predicting performance from self-compassion or well-being.

Response Variable		R^2^_adj_	β	f^2^	*t*	*p*	95% CI	DW
Self-Compassion	‘Perceived’ Performance	0.03	−0.20	0.04	−1.91	0.06	−0.81; 0.02	2.25
	‘Actual’ Performance	0.02	−0.17	0.03	−1.60	0.11	−2.12; 0.23	2.15
Well-Being	‘Perceived’ Performance	0.01	−0.16	0.03	−1.45	0.15	−0.79; 0.12	2.18
	‘Actual’Performance	0.00	−0.01	0.00	−0.12	0.91	−1.38; 1.23	2.09

Note: R^2^_adj_ = Adjusted R-squared; β = standardized beta-coefficient; f^2^ = estimate of effect size; *t* = *t*-test statistic; *p* = significance of *t*-test statistic; 95% CI = ninety-five percent confidence interval; DW = Durbin–Watson statistic. The sample size was N = 87 (self-compassion) and n = 86 (well-being).

**Table 3:** Hierarchical regression analyses examining the influence of well-being beyond self-compassion on performance indicators.

Response Variable	Explanatory Variable	B	SE_B_	95% CI_B_	β	*t*	r_s_	RPI	R^2^	R^2^_adj_	ΔR^2^
‘Perceived’ Performance	Step 1								0.04	0.03	
	Self-compassion	−0.39	0.21	−0.81; 0.03	−0.20	−1.87	1.00	1.00			
	Step 2								0.05	0.03	0.01
	Self-compassion	−0.33	0.22	−0.77; 0.12	−0.17	−1.46	−0.91	0.68 ^a^			
	Well-being	−0.22	0.24	−0.70; 0.27	−0.10	−0.88	−0.73	0.32 ^a^			
‘Actual’ Performance	Step 1								0.03	0.03	
	Self-compassion	−1.01	0.59	−2.19; 0.17	−0.18	−1.70	−0.93	1.02			
	Step 2								0.04	0.01	0.00
	Self-compassion	−1.12	0.63	−2.37; 0.14	−0.20	−1.76	−0.89	0.85 ^a^			
	Well-being	0.34	0.69	−1.04; 1.71	0.06	0.49	0.21	0.06			

Note: B = unstandardized beta-coefficient; SE_B_ = standard error of unstandardized beta-coefficient; 95% CI_B_ = ninety-five percent confidence interval around the point estimate for the unstandardized beta-coefficient; β = standardized beta-coefficient; *t* = *t*-test statistic; r_s_ = structure coefficient; RPI = Relative Pratt Index; ^a^ meaningful predictor (*RPI* cut-off = 0.25). R^2^ = Coefficient of Determination. *R^2^_adj_* = Adjusted R-square; ΔR^2^ = change in R-square. The sample size was N = 87 (self-compassion) and n = 86 (well-being) per explanatory variable.

## Data Availability

Data are available upon request from dmack@brocku.ca.

## Supplementary Materials

The following supporting information can be downloaded at https://www.mdpi.com/article/10.3390/sports12110300/s1, Table S1: Comparison between pilot and main study participants across general and golf demographic variables; Table S2: Bivariate correlations between male and female participants; Figure S1: The relationship between self-compassion scores and ‘perceived’ performance by gender; Figure S2: The relationship between self-compassion scores and ‘actual’ performance by gender; Figure S3: The relationship between well-being scores and ‘perceived’ performance by gender; Figure S4: The relationship between well-being scores and ‘actual’ performance by gender.
