# Supplementary material for: Treating Yourself in a Fairway: Examining the Contribution of Self-Compassion and Well-Being on Performance in a Putting Task
Source: Sports (Basel). 2024 Nov 5;12(11):300. doi: 10.3390/sports12110300 (PMC11598489; doi:10.3390/sports12110300)
Supplement: Supplementary file 1 [file sports-12-00300-s001.zip › sports-3246250-supplementary.pdf]

Supplemental Materials

Pilot Study

Five golfers were recruited to perform the putting task. Inclusion criteria for selection mirrored that for participants of the main study. Participants included in the pilot phase were not eligible to participate in the main study. They were entered into a draw for a chance to win one $50 gift card. Participants volunteering for this phase were asked to complete the general and golf-specific demographic questionnaires, the familiarization and putting task, the measure of perceived performance and challenge. The purpose of this pilot task was to offer insight into the golf putting task adopted in this study on performance and to standardize researcher protocol.

The appropriate parametric (*t*-test) or non-parametric (χ^2) test was conducted to examine differences between pilot and main study participants on demographic variables (see Table S1). A large effect size for the number of rounds played in 2022 was found (d = 0.75), yet statistical significance was not achieved (i.e., p = .11). No other statistically significant differences between pilot and main study participants were found ($p > .05$) with effect sizes ranging from d = .01 to .39.

Environmental Conditions and Stimpmeter Readings

The average weather across the 28-day main study data collection period was 23.51 degrees Celsius (*SD* = 3.36; *Range* = 15 – 28). Precipitation fell on one day of data collection with humidity averaging 60.54% (*SD* = 15.29%; *Range* = 26% - 84%). The average stimpmeter reading was 7.75 feet (*SD* = 0.70; *Range* = 6.09 - 9.08) which is within the range of acceptability to measure putting performance (Turner et al., 2018).

Main Study: Consideration of Gender Differences

For demographic variables, only number of years playing golf differed significantly (p = .01; d = 1.26) with males reporting playing longer ($M = 31.60_{\text{years}}$; $SD = 15.89_{\text{years}}$) than females ($M_{years}$ = 12.00; SD_{years} = 9.46). All other demographic variables were non-significant ($p > .05$) with corresponding d values in the weak to small range (Cohen, 1992). When looking at main study variables, no statistically significant differences ($p > .05$) emerged with estimates of practical significance ranging from $d_{perceived.\ performance} = .15$ to $d_{actual.performance} = .53$.

Gender differences in terms of direction and or magnitude appeared for Pearson bivariate correlations for main study variables (see Table S2). Therefore, it may be that gender moderated many of the relationships between study variables and was further reinforced through inspection of the scatterplots (see Figures S1 – S4) for three of the four apriori planned simple linear regression analyses. Based on all information presented, it was decided to remove female participants from the larger analyses of this study.

Table S1

Comparison between Pilot and Main Study Participants Across General and Golf Demographic Variables

	Pilot		Main					
Variable	*M*	*SD*	*M*	*SD*	*t*	*df*	*p*	*d*
Age (years)	52.40	18.19	54.94	15.37	-0.36	89.00	0.72	0.16
How many years have you been playing golf?	37.80	17.11	31.60	15.89	0.84	75.00	0.40	0.39
How many rounds of golf have you played in 2022?	43.40	40.96	27.73	19.53	0.85	4.11	0.11	0.75
If yes, please provide your Golf Canada Handicap	9.10	4.88	9.93	6.31	-0.22	23.00	0.83	0.13
How important is putting to golf performance?	4.40	0.55	4.62	0.71	-0.67	89.00	0.50	0.31
How important is putting to your golf performance?	4.60	0.55	4.57	0.64	0.10	89.00	0.92	0.05
How important is putting to you as a golfer?	4.60	0.55	4.60	0.60	0.02	89.00	0.99	0.01
Actual Performance	7.80	2.28	7.86	3.10	-0.04	90.00	0.97	0.02
Perceived Performance	2.40	0.55	2.56	1.10	-0.33	90.00	0.74	0.15
How challenging was the putting task you performed today?	1.80	1.10	1.80	1.01	0.01	90.00	0.99	0.01
	%		%			χ^2	*p*	*phi*
Self-Identified Ethnicity						18.01	0.01	0.44
Arab			1.10					
Asian			2.30					

Black	20.00	1.10			
South Asian		1.10			
White	80.00	88.50			
Indigenous		4.60			
Other		1.10			
Level of Competition			6.38	0.04	0.27
Recreational	60.00	88.50			
Competition-Amateur	40.00	6.90			
Competition-CPGA/PGA Professional		2.30			
Are you a Golf Canada Member?			3.01	0.08	0.18
Yes	60.00	24.10			
No	40.00	73.60			
Do you have any injuries that may impact your putting performance today?			0.58	0.45	0.08
Yes		10.30			
No	100.00	88.50			

Note. *M* = Mean; *SD* = Standard deviation; *t* = t-test statistic; χ^2 = *Chi-square statistic; p* = significance of inferential test; *d* = effect size (Cohen, 1992); *phi* = phi coefficient (Grissom & Kim, 2005). The sample size for the pilot study was *n* = 5 and ranged from 72 to 87 for the main study depending upon variable.

Table S2

Bivariate correlations between male and female participants

	1	2	3	4
1. Self-Compassion	-	0.48	0.40	-0.17
2. Well-Being	0.34	-	0.84	0.23
3.Actual Performance	-0.17	0.04	-	0.70
4. Perceived Performance	-0.20	-0.16	0.71	-

Note: All *r's* expressed in the upper diagonal of the matrix are bivariate (Pearson) coefficients for female participants, $n = 5$. All *r's* expressed in the lower diagonal of the matrix are bivariate (Pearson) coefficients for male participants, $N = 87$.

Figure S1

The relationship between self-compassion scores and perceived performance by gender

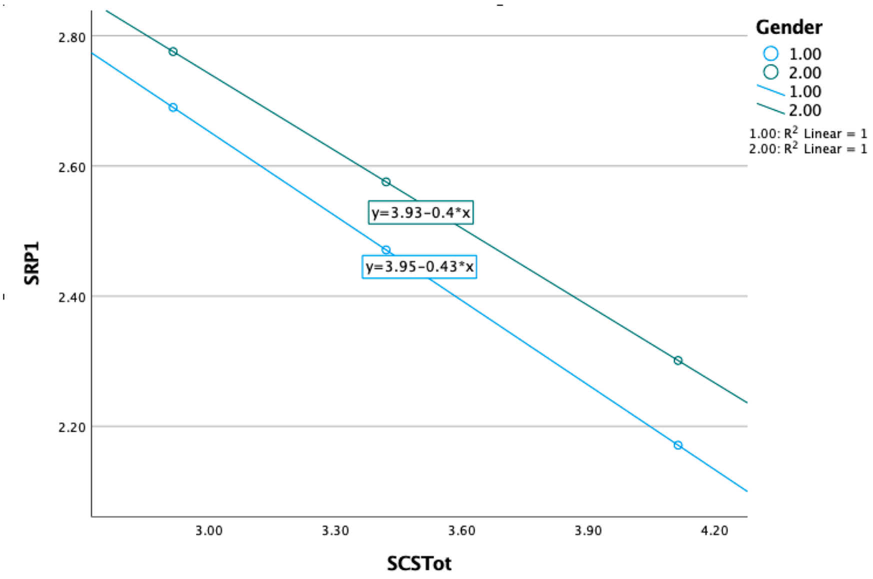

Note: SRP1 = Perceived performance; SCSTot = Self-compassion; 1 = female; 2 = male

Figure S2

The relationship between self-compassion scores and actual performance by gender

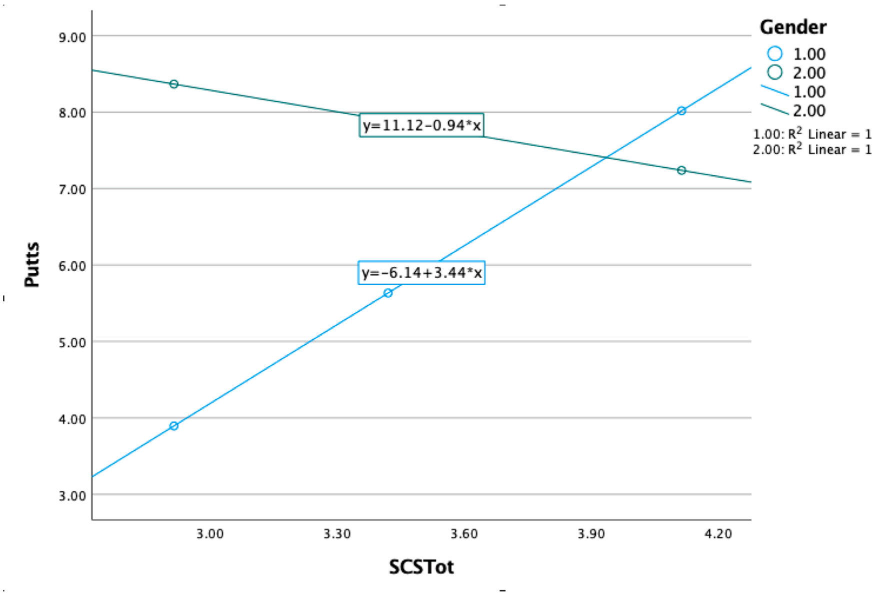

Note: Putts = Actual performance; SCSTot = Self-compassion; 1 = female; 2 = male

Figure S3

The relationship between well-being scores and perceived performance by gender

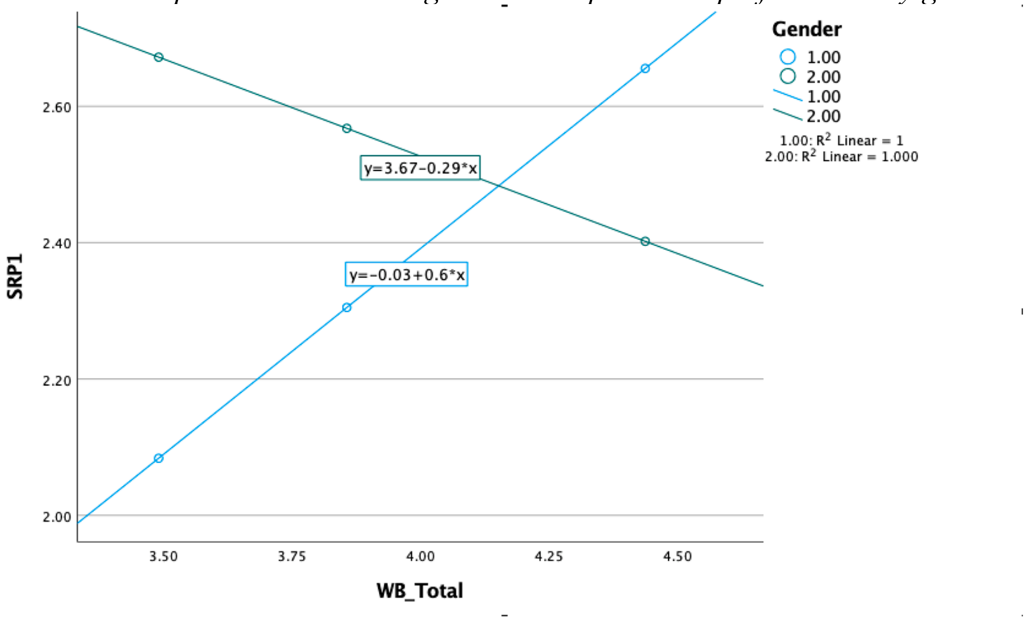

Note: SRP1 = Perceived performance; WB Total = WEMWBS Total; 1 = female; 2 = male

Figure S4

The relationship between well-being scores and actual performance by gender

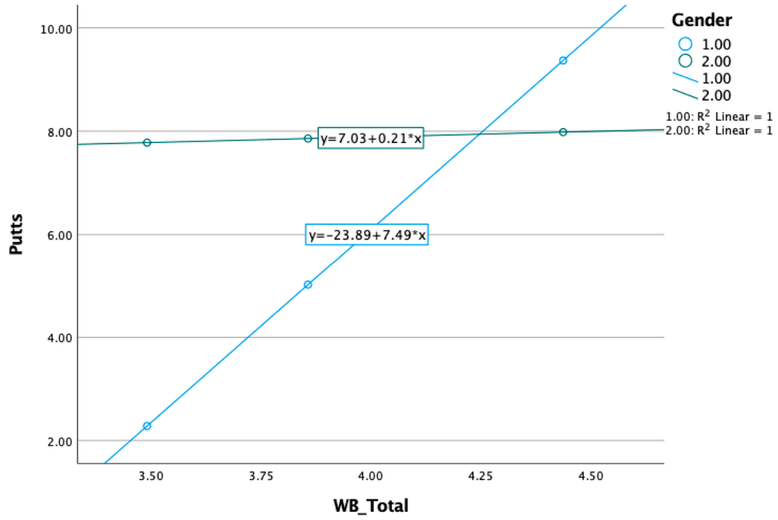

Note: Putts = Actual performance; WB Total = WEMWBS Total; 1 = female; 2 = male
